# From Portuguese Colonial Representations to Racist Endorsement: Investigating Correlational and Causal Paths

**DOI:** 10.5334/irsp.1110

**Published:** 2026-05-12

**Authors:** Felix Meuer, Rita Guerra, Filipa Madeira, Joaquim Pires Valentim

**Affiliations:** 1 University of Coimbra, Faculty of Psychology and Educational Sciences, Coimbra, Portugal; 2 Iscte - Instituto Universitário de Lisboa, Centro de Investigação e Intervenção Social (CIS-Iscte), Lisboa, Portugal; 3 Psychology Department, Faculty of Human Sciences, Catholic University of Portugal, Lisbon, Portugal; 4 Institute of Social Sciences, University of Lisbon, Lisbon, Portugal; 5 University of Coimbra, Faculty of Psychology and Educational Sciences, Center for Research in Neuropsychology and Cognitive and Behavioral Intervention, Coimbra, Portugal

**Keywords:** social representations of history, Luso-tropicalism, Intergroup relations, biological racism, cultural racism

## Abstract

Dominant social representations of history play a central role in shaping how societies interpret the past and regulate intergroup relations in the present. Yet empirical evidence on how historical narratives causally influence racist beliefs remains limited. Drawing on the Social Representations of History framework, the present research examines how dominant and counter-representations of colonial history relate to the endorsement of biological and cultural racism. Focusing on the Portuguese context, where colonial history is often narrated through a positive and benevolent lens grounded in the ideology of Luso-tropicalism—the belief that Portuguese colonialism was uniquely tolerant and benign—we investigate both correlational and causal pathways linking these representations to racist attitudes. In Study 1 (*N* = 216), using data from CRONOS-2, part of the European Social Survey Round 10, we show that endorsement of Luso-tropicalist beliefs predicts higher levels of both biological and cultural racism, above and beyond age and education. In Study 2 (*N* = 220), we experimentally manipulated representations of colonial history using three conditions: a positive representation derived from a textbook, a negative representation, and a neutral control. Results indicate that biological racism was significantly lower when colonial history was presented in a negative frame compared to a combined positive and neutral representation, while no significant differences emerged for cultural racism. Together, findings from Study 1 and Study 2 provide novel evidence that counter, negative representations of colonial history can attenuate racist beliefs. By integrating correlational and experimental evidence, this research contributes to broader debates on social representations of history as political projects with enduring consequences for contemporary intergroup relations.

The study of historical memory has gained increasing attention in social psychology (Babínska & Licata, 2026), anchored in the proposal that selected historical narratives are rooted in socially constructed hierarchies of power shaping current intergroup relations (e.g., Said, 1993; Babínska & Licata, 2026). The social standing of a group – for example whether dominant or marginalized – determines whether a historical narrative is heard or silenced, exemplifying the idea that historical narratives are identity forming and constitute an essential determinant of the imagined concept of nationhood (e.g., Hobsbawm, 1990). Therefore, individuals are exposed to fragments of history, such as in the educational context, where young people fail to engage with multiple perspectives of a group’s past, and instead engage with history as the ultimate truth (e.g., Van Nieuwenhuyse, 2022). To study historical narratives from a social psychological perspective, social representations theory offers a common tool to better understand how history telling takes part in processes of meaning making (Van Nieuwenhuyse & Valentim, 2018). According to this framework, historical memory is understood as narratives about the past that are socially constructed, encompassing group norms and values (e.g., Sakki, 2025), thereby shaping intergroup attitudes.

The current research builds on social representations theory to better understand how selected historical narratives shape racist attitudes. To do so, we looked at the Portuguese case, a historical context determined by decades of exploitation and legitimizing practices to justify colonial invasions. In Study 1 we examined how a hegemonic social representation about benevolent colonialism (i.e., Luso-tropicalism) predicts racist endorsement using representative data from the CRONOS-2, a pillar of the European Social Survey 10 (ESS10; Sikt, 2020). In Study 2, we conducted an experiment retrieved from educational material to test the causal impact of a negative representation of colonialism (vs. positive and control) on racism endorsement. Together the two studies extend existing research in two ways: 1) understanding the connection between selected historical narratives and the endorsement of racism combining correlational and experimental evidence; 2) using the educational context, in form of textbook narratives as an everyday example of how historical narratives shape racism.

## Social Representations Theory and the Case of History

Social representation theory explains how individuals in different social contexts communicate about social phenomena, defining social representations as a ‘system of values, ideas, and practices’ that enable communication and help individuals to make sense of the world (Moscovici, 1961/2008). While Moscovici emphasized the communicative and sense-making functions of social representations, later developments of the theory have highlighted their ideological role in legitimizing group interests and social inequalities (Howarth, 2006). In line with this perspective, social representations are understood in the present paper as a dynamic process that shapes how individuals act within their social environments and interact with others (Howarth, 2006).

A social order comprises a plurality of social representations, among which hegemonic representations constitute the dominant forms of knowledge accepted within a given social group. The production and dissemination of these representations are therefore embedded in existing power structures, which determine who acts as an agent of knowledge and who remains a passive recipient. Counter-representations that challenge dominant narratives are often rejected, contributing to the reproduction of discriminatory social practices (Howarth, 2006). From this perspective, social representations are not merely shared understandings of the social world, but tools through which social hierarchies and intergroup inequalities are produced and maintained.

In line with social representations theory, understandings of collective identity are closely tied to how groups relate to their past. To understand and perceive who we are, individuals and groups often look back to historical narratives that define the origins, values, and norms of the social group (Liu & Hilton, 2005). By endorsing hegemonic narratives of a group’s history, actions in the present and future are morally legitimized, providing a framework for evaluating intergroup relations and inequalities as justified or unjustified. In this sense, historical representations constitute forms of collective memory that contribute to group cohesion and continuity (Bar-Tal, 2014). However, these representations are not static. Moscovici (1961; 2008) argued that social representations are generated and transformed through mechanisms of social communication, such as propagation and propaganda, that are inherently selective. Dominant groups are particularly well positioned to mobilize these communicative processes to create or maintain their versions of history, thereby institutionalizing them in ways that maintain the existing social hierarchy. As a consequence, hegemonic discourse of history rejects counternarratives that could threaten the national ingroup (Howarth, 2006). When embedded in institutional contexts such as education, hegemonic representations of history acquire a taken-for-granted status, making alternative interpretations less visible and less legitimate (Liu & Hilton, 2005).

In the case of colonial history and the domination of European colonial powers, hegemonic narratives successfully silence the reality of domination (e.g., Bouchat et al., 2023). Colonial narratives continuously depict Europeans at a higher social position compared to former colonized peoples, using strategies such as that of the indigenous ‘noble savage,’ which depicts native individuals as child-like beings ruled by instinct, effectively stripping them of rationality (Jahoda, 1999). Such representations illustrate how colonial narratives do not merely describe historical relations but actively construct racialized hierarchies that continue to inform contemporary perceptions of outgroups.

### Social Representations of History: From Common Sense to Attitude Formation

Representations of history play a central role in transforming socially shared interpretations of the past into organizing world views to navigate the social world. They naturalize particular beliefs and norms by embedding them, thereby creating objective truths. To explain this phenomenon, Liu and Hilton (2005) introduced the concept of historical charters, defined as dominant historical representations composed of selective elements of a group’s history. Empirical work from New Zealand, where bicultural relations between Māori and Pākehā structure social relations, illustrates how such historical charters shape contemporary intergroup attitudes. Liu and colleagues (2014) examined the Treaty of Waitangi as a foundational historical event with charter status. Although exposure to this historical event did not alter average levels of bicultural attitudes, individuals’ interpretations of the event’s meaning and relevance predicted such attitudes. These findings demonstrate that historical representations with charter status incorporate specific attitudinal orientations that become psychologically consequential for intergroup relations. Complementary evidence comes from the Belgian context, where colonial history is frequently narrated in terms of development and exploitation. Lastrego and colleagues (2023) showed that exposing participants to a positive historical representation based on fostering development to colonized regions was associated with more negative attitudes toward Congolese individuals, less willingness to compensate for historical misdeeds, and higher endorsement of modern racism. A historical representation about the exploitative nature of Belgian colonialism had the opposite effect (Lastrego, Magonet, & Licata, 2023). These findings are particularly consequential given that dominant groups tend to preferentially remember and endorse historical narratives that portray the ingroup in positive terms, thereby reinforcing a sense of moral superiority and positive ingroup distinctiveness in intergroup and international comparisons (Bouchat et al., 2023).

Taken together, these findings highlight two key theoretical implications. First, social representations of history that acquire the status of a historical charter are decisive in shaping intergroup relations, by embedding specific attitudes within dominant historical narratives. Second, hegemonic representations that emphasize benevolence and moral superiority are associated with the reinforcement of negative outgroup attitudes. Building on this work, the present research examines whether similar dynamics operate in the Portuguese context and whether dominant (i.e., positive) and counter-hegemonic representations (i.e., negative) of colonial history are linked to the endorsement of racist beliefs.

### Representations of History and Outgroup Attitudes: The Portuguese Case

The Portuguese case offers a theoretically informative context for examining how social representations of history shape contemporary racist attitudes. Former Portuguese colonies only gained independence after the prolonged and violent colonial wars in 1974. Despite late processes of decolonization, hegemonic social representations in Portugal portray Portuguese society as relatively free from racism and discrimination (Castelo, 1999).

However, empirical evidence challenges this narrative. Data from the European Social Survey indicates that biological racism which legitimizes social hierarchies through presumed biological differences, was endorsed by approximately 50% of Portuguese respondents, a proportion significantly higher than the European average (Vala & Pereira, 2018). At the same time, recent survey evidence points to widespread endorsement of benevolent representations of Portuguese colonial history, with more than half of the Portuguese population agreeing that colonialism was largely positive and essential for the development of the former African colonies (Madeira et al., 2023; Valentim & Heleno, 2018). Taken together, these findings reveal a striking paradox: high levels of adherence to benevolent historical representations coexist with elevated levels of racism.

From a Social Representations perspective, this pattern suggests that myths of Portuguese colonial exceptionalism—actively promoted during the Estado Novo regime—continue to structure collective memory more than 50 years after the end of the dictatorship. One of the central ideological frameworks underlying this process is Luso-tropicalism (Castelo, 1999), a social theory originally proposed by Brazilian sociologist Gilberto Freyre (1933) and later transformed into a dominant social representation portraying Portuguese colonialism as uniquely tolerant, harmonious, and non-racist. During the Estado Novo (1933–1974), the regime strategically mobilized Luso-tropicalism as propaganda to legitimize colonial rule in the face of increasing international pressure for decolonization. Although the dictatorship ended decades ago, Luso-tropicalist beliefs remain widely endorsed in contemporary Portuguese society (Madeira et al., 2023; Valentim & Heleno, 2018).

Importantly, the dissemination of Luso-tropicalist ideas did not occur solely at the level of political discourse but was also institutionalized through education. A political core of Luso-tropicalist ideology was embedded in history textbooks during the Estado Novo (Castelo, 1999), and traces of this benevolent narrative persist in more recent editions, symbolically benefiting the Portuguese ingroup. From a social representation perspective, textbooks play a central role in the production of common sense by circulating scientific, commonsensical, and ideological forms of knowledge that provide the raw material for broader social representations to develop and endure (Van Nieuwenhuyse & Valentim, 2018). As institutionalized narratives aligned with state curricula, history textbooks contribute to the formation of social identity and national belonging, often through increasingly nationalist ways of narrating the past (Carretero et al., 2011; Sakki, 2025).

Consistent with this perspective, research on Portuguese textbooks shows that colonial history is predominantly framed positively. Studies indicate that narratives of the so-called ‘discoveries,’ particularly in relation to Brazil, emphasize economic and political interests while downplaying colonial power relations (Soares & Jesuíno, 2004). Textbooks used in key-stage 3 education (years 7–9) depoliticize colonial power-relations and narrate Portuguese colonial history through a Eurocentric lens (Araújo & Maeso, 2012). Although important changes occurred between 1957 and 2015, contemporary textbooks still systematically minimize Portuguese atrocities and attribute positive moral traits to the colonizer. Whereas certain dimensions of Luso-tropicalism appear less frequently in textbooks, overall representations of colonial history remain predominantly benevolent in the educational materials analyzed (Valentim & Miguel, 2018).

Against this backdrop, racism constitutes a particularly relevant outcome for examining the attitudinal consequences of dominant representations of colonial history. Racism as an ideology historically rooted in colonialism, asserts that human beings are divided into naturally distinct ‘races’ with different attributes—a framework developed to legitimize European colonial domination under the guise of a ‘civilizing mission’ (Bethencourt, 2013). From this perspective, benevolent representations of colonial history that align with Luso-tropicalist worldviews may contribute to the normalization and legitimation of both biological and cultural forms of racism. Biological racism denotes the belief that human groups are organized into biologically distinct categories whose differences are innate. In contrast, cultural racism denotes the belief that groups differ in fixed cultural attributes, allowing for hierarchical evaluations and exclusionary attitudes without explicitly invoking biological inferiority (Vala & Pereira, 2018). Accordingly, racist attitudes can be understood as embedded within the historical charter of positive representations of Portuguese colonialism, particularly as these narratives continue to be reproduced in educational contexts. Building on this reasoning, the present research examines how dominant representations of Portuguese colonial history—and exposure to alternative, counter-representational narratives—relate to the endorsement of biological and cultural racism.

### Present Research

Building on the Social Representations of History framework (Liu & Hilton, 2005) and research on Luso-tropicalism as a dominant ideological representation of Portuguese colonial history (Valentim & Heleno, 2018), the present research examines how representations of colonial history are linked to the endorsement of racist beliefs in Portugal. Across two complementary studies, we investigate both correlational and causal pathways connecting representations of the colonial past to biological and cultural racism.

In Study 1, we examine whether endorsement of Luso-tropicalist beliefs predicts higher levels of biological and cultural racism. Drawing on evidence that historical representations with charter status embed intergroup attitudes (Liu, Sibley, & Huang, 2014), as well as prior research linking Luso-tropicalism to prejudice in Portugal (Valentim & Heleno, 2018), we hypothesize that endorsement of Luso-tropicalism will be associated with higher levels of both biological and cultural racism, above and beyond age and level of education (H1).

Study 2 extends these findings by experimentally testing the causal impact of exposure to different representations of Portuguese colonial history. Using educational materials, we manipulate colonial narratives presented in positive, negative (counter-representational), and neutral forms. While Luso-tropicalism constitutes a multidimensional ideological construct, the positive condition targets discursive representations of colonial history that are compatible with Luso-tropicalist ideology, particularly those emphasizing benevolence and moral exceptionalism. Such narratives frequently appear in institutionalized educational narratives, wherefore fragments of textbooks were used to create this manipulation (Valentim & Miguel, 2018). Consistent with findings from the Belgian context (Lastrego, Magonet, & Licata, 2023), we hypothesize that participants exposed to a negative colonial representation will report lower levels of biological and cultural racism compared to those exposed to positive and neutral representations (H2). From a social representation perspective, the positive representation is expected to function as historical charters (Liu, Sibley, & Huang, 2014)—that is, normative representations whose associated attitudes are already widely shared within the dominant group, similarly to a control condition. Given this theoretical rationale, positive and control conditions are pooled for hypothesis testing. Overall, exposure to a negative representation of colonial history highlighting violence and exploitation is expected to operate as a counter-representation capable of challenging racist endorsement.

## Study 1

### Method

#### Sample

The present study included 216 residents from Portugal, aged 18–80 years (*M* = 44.28, *SD* = 13.92). Of these, 93 (43.1%) identified as male and 123 (56.9%) as female. Concerning educational attainment, 49.6% of the participants completed up to secondary education, whereas 50.4% completed advanced vocational or university degree courses.

#### Procedure

This study used data from the Cross-National Online Survey 2 (CRONOS-2), a cross-national panel implemented in 12 European countries, including Portugal, as a complement to the European Social Survey Round 10 (ESS10; Sikt, 2020). In Portugal, the CRONOS-2 survey module on ‘Antecedents and Consequences of Luso-Tropicalism’ was selected through an open national competition. Respondents from Round 10 of the ESS (2020/2022) in Portugal who had Internet access were invited to participate in the CRONOS-2 panel. All participants were offered incentives worth EUR 5 for their participation. Data was administered using Qualtrics survey software and collected between January 2022 and February 2023. For the present analyses, we used CRONOS-2 measures of Luso-tropicalism, biological racism, and cultural racism.

### Measures

All items were measured on a 5-point Likert scale (1 = strongly disagree to 5 = strongly agree).

#### Luso-Tropicalism

Informed by previous research on social representations of colonialism (Licata et al., 2018; Lima-Nunes, Pereira, & Correia, 2013; Liu & Hilton, 2005; Valentim & Heleno, 2018) 12 items were used to assess participants’ agreement with dimensions of social representations of Portuguese colonialism. The dimensions include: Racial Denial (α = .77; e.g.: ‘Compared to other European countries, there is less racism in Portugal’), Symbolic Inclusion (α = .41; e.g.: ‘The Portuguese and people from previously colonized nations should be considered as a single linguistic and cultural community’), Miscegenation (α = .85; e.g.: ‘Portuguese colonial history was characterized by the ability of the Portuguese to mix with the colonized peoples’), and Development (α = .92; e.g.: ‘Portuguese colonialism was fundamental to the economic development of the former colonies’). We created a composite score of all items, where higher scores meant higher endorsement to Luso-tropicalism (α = .80).

#### Biological Racism

Biological racism was measured using two items: ‘Thinking about various groups of people, there are racial and ethnic groups that are, by nature, less intelligent than others’ and ‘There are racial and ethnic groups that are, by nature, harder-working than others’. Both items were found to be strongly positively correlated, *r*(377) = .71, *p* < .001. A composite score was created where higher scores indicate higher endorsement to biological racism.

#### Cultural Racism

Cultural racism was measured using two items: ‘Some cultures are clearly superior to others’ and ‘Thinking about today’s world, some cultures are much better than others’. Both items were found to be strongly positively correlated, *r*(377) = .60, *p* < .001. A composite score was created where higher scores indicated higher endorsement to cultural racism.

### Results

#### Preliminary Results

Statistical analyses were conducted using IBM SPSS Statistics (version 26). Correlations are presented in the appendices (see Appendix 1 through the link provided in the data accessibility statement). As expected, Luso-tropicalism was positively associated with both cultural and biological racism. Moreover, age was positively correlated with biological racism and Luso-tropicalism, but not with cultural racism. Education was negatively correlated with biological racism and cultural racism, but not with Luso-tropicalism. No significant correlations were found for income and gender. Hence, it was decided not to include income and gender for the main analysis.

#### Main Analysis

To test H1, we conducted two multiple linear regression analyses (see Table 1). In both models, the predictors were Luso-tropicalism, age, and education, with outcomes being biological racism and cultural racism, respectively. For biological racism, the overall model was significant and Luso-tropicalism significantly predicted biological racism endorsement, even after controlling for age and education. Similar results were found for cultural racism. Here, the overall model was significant as well, and Luso-tropicalism significantly predicted cultural racism endorsement, over and above age and education. These findings support H1 and suggest that endorsement of Luso-tropical beliefs is uniquely associated with higher levels of both biological and cultural racism, independently of key sociodemographic factors.

**Table 1:** Regression models predicting biological and cultural racism.


BIOLOGICAL RACISM						CULTURAL RACISM				

EFFECT	ESTIMATE	*SE*	95% CI		*p*	ESTIMATE	*SE*	95% CI		*p*
	
			EFFECT					EFFECT		
	
			*LL*	*UL*				*LL*	*UL*	

Intercept	1.95	.44	1.08	2.81	<.001	1.89	.5	.91	2.88	<.001

Luso-tropicalism	**.28**	.12	.04	.52	.024	**.41**	.14	.14	.69	.004

Age	.01	.01	0	.02	.13	0	.01	-.01	.02	.47

Education	**–.17**	.04	–.24	–.1	<.001	**–.17**	.04	–.25	–.09	<.001

*R^2^* = .16, *F*(3. 212) = 13.64, *p* < .001						*R^2^* = .13, *F*(3, 212) = 11.31, *p* < .001				


## Study 2

### Method

#### Sample

We targeted a small-to-medium effect size of *f* = 0.21, informed by the empirical average effect size (*r* = .21) reported in the meta-analytic review by Richard et al. (2003). To align this estimate with an omnibus one-way ANOVA, we converted *r* to Cohen’s *f* (*f* = 0.21). An a priori power analysis conducted in G*Power (Faul et al., 2009) assuming α = .05, power (1–β = .80), and three conditions, indicated that approximately 222 participants would be required.

Eligibility criteria included a minimum age of 18 years and Portuguese nationality. We collected 319 responses; 99 were excluded because questionnaires were not properly completed (*n* = 83) or because participants were not Portuguese nationals (*n* = 16). The final sample consisted of 220 Portuguese participants randomly assigned to three conditions (negative, positive, and control). Participants had a mean age of 33.93 years (*SD* = 13.8, range: 18–89). Most participants identified as women (63.6%), were employed (60%), held a bachelor’s or postgraduate degree (67.2%), and reported being financially comfortable or able to make ends meet (75.9%) (see Appendix 2).

#### Procedure

A local ethics committee approved all materials of this study (32/2023). Data was collected online using Qualtrics (2020). Participants were recruited via e-mail, social media, using QR-Code distribution, convenience sampling, or directly approached on the streets of Lisbon. Participants were first presented with informed consent, which included the researchers’ contact information, the study’s duration, and information on the voluntary, anonymous, and confidential nature of participation. Once accepted, participants answered the demographic questions. Participants were then randomly assigned to one of three conditions. After being exposed to the manipulation, participants answered all measures of interest: biological racism and cultural racism, presented in random order. At the end, participants were debriefed and thanked for participation.

### Materials and Measures

#### General Demographics

Participants indicated their age, level of education, employment status, gender identification, nationality, country of birth, satisfaction with present income, and political orientation.

#### Manipulations

In all experimental conditions, participants were informed that they would take part in a study about current debates on how history should be transmitted in educational contexts. They were instructed to carefully read a short text and were randomly assigned to one of three conditions: positive representation of colonial history, negative representation of colonial history, or control. In the positive representation condition, participants read an excerpt retrieved from passages of a Portuguese history textbook used in primary education (Alves & Jesus, 2019). The passages selected for the manipulation conveyed a predominantly benevolent narrative of Portuguese colonial history, emphasizing themes of exploration, encounter, and development. The textbook was designed for the 5th grade and written by one of the major Portuguese publishers, Porto Editora. In Portugal, the ministry of education describes a mandatory six-year adoption cycle for textbook use. But even after these six years, many schools are advised to finish their stock before introducing new books. This means that, despite its 2019 publication year, the textbook is still used in Portuguese schools. The textbook passages retrieved from this textbook were edited for a fluent text to emerge. In the negative representation condition, the same base text was systematically adapted to emphasize the violent, exploitative, and coercive aspects of Portuguese colonialism. The overall structure, length, and style of the text were kept constant across conditions, with changes restricted to the semantic content relevant to the historical evaluation. In the control condition, participants read a neutral informational text unrelated to history or intergroup relations, describing biological characteristics of frogs (Ramalho, 2023; see link provided in the data accessibility statement). The three texts were approximately equivalent in length (*M* = 220.67 words).

#### Biological and Cultural Racism

Biological racism was measured using two items adapted from the ‘European Social Survey 9’ (ESS9; Sikt, 2018): ‘Some races or ethnic groups are born less intelligent than others,’ and ‘Some races or ethnic groups are born harder working than others’ (5-point Likert scale, 1 = strongly disagree to 5 = strongly agree). We created a composite score, where higher scores indicate higher endorsement to biological racism (*r* (206) = .63, *p* < .001). For cultural racism, we also adapted two items from ESS9 and participants indicated their agreement (5-point Likert scale, 1 = strongly disagree to 5 = strongly agree): ‘Some cultures are, by nature, more civilized than others,’ and ‘Humanity is divided into very different cultures.’ The two items were weakly correlated (*r* (208) = .25, *p* < .001), thus, no composite score was created. We tested our hypotheses using ‘Some cultures are, by nature, more civilized than others.’ We chose this measurement based on the facial validity of the item to ensure it aligns with what we wanted to measure. A focus on some cultures being more civilized than others better reflected the notion of an existent social hierarchy, compared to a statement that only emphasized the existence of cultural differences. For the chosen item, a higher score indicates more endorsement to cultural racism.

#### Manipulation Check

Participants indicated on a 5-point Likert scale (1 = very negative to 5 = very positive) whether they felt the text they read at the beginning of the study portrayed a positive or a negative representation of Portuguese history (‘In the excerpts you just read, how was Portuguese history presented to the readers?’).

### Results

#### Preliminary Results

Statistical analyses were conducted using IBM SPSS Statistics (version 26). We first conducted an independent sample *t*-test to evaluate the manipulation’s efficacy. In line with what we expected, participants in the positive representation condition (*M* = 3.45, *SD* = 1.02) rated the excerpt as significantly more positive than those in the negative representation condition (*M* = 2.30, *SD* = .89) regarding the way Portuguese history was represented, *t*(124) = –6.8, *p* < .001).

#### Main Analysis

To examine whether historical representations of colonialism influenced racist attitudes, we conducted two one-way between-subject ANOVAs, with biological racism and cultural racism as the dependent variables. Participants were randomly assigned to one of three conditions: positive representation of colonialism, negative representation of colonialism, or control. In line with the historical charter framework, we computed two orthogonal, theory-driven contrasts: (1) a contrast comparing the negative representation condition against the pooled positive and control conditions, and (2) a contrast comparing the positive representation condition against the control condition. The contrast of interest compares the negative condition with the pooled control and positive conditions to test H2. For biological racism, the effect of condition was significant, *F*(2, 205) = 4.37, *p* = .01, η*²* = .04. Consistent with H2, contrasts showed that biological racism was lower in the negative condition (*M* = 1.63, *SD* = .93) compared to the pooled positive (*M* = 1.8, *SD* = .96) and control (*M* = 2.13, *SD* = 1.1) condition, *t*(205) = –2.76, *p* = .01. No significant difference was found between the control condition and the positive condition, *t*(205) = –1, *p* = .32.

For cultural racism, no significant effect of condition was found, *F*(2, 207) = .41, *p* = .66, η*²* = .004. Contrasts indicated no significant difference between the negative condition (*M* = 2.7, *SD* = 1.41) compared to the pooled positive (*M* = 2.84, *SD* = 1.3) and control condition (*M* = 2.9, *SD* = 1.33), *t*(207) = –.66, *p* = .51). Moreover, contrast 2 showed no significant difference between the positive and the control condition, *t*(207) = –.62, *p* = .54.

Additional exploratory analysis (i.e., simple contrast) showed no differences when comparing the positive (*M_biologicalracsim_* = 1.8, *SD* = .96; *M_culturalracism_* = 2.84, *SD* = .1.29) and negative (*M_biologicalracism_* = 1.63, *SD* = .93; *M_culturalracism_* = 2.7, *SD* = .1.41) conditions for either biological racism, (*t*(138) = –1.06, *p* = .29) or cultural racism (*t*(139) = –.61, *p* = .54). All in all, results provide partial support for the hypothesis that exposure to negative representations of colonialism impacts racism endorsement: biological racism was significantly less endorsed by participants exposed to negative condition compared to those in the pooled positive and control, but no differences were found for cultural racism. Overall, there were no differences between the positive and neutral conditions.

## General Discussion

The present research investigated how historical representations of colonialism impact the expression of racist beliefs in Portugal. Across two studies, the findings converge on a central pattern: dominant, benevolent representations of Portuguese colonialism are associated with higher endorsement of racist beliefs (Study 1) and exposure to a critical, counter-hegemonic representation of colonial history reduces endorsement of biological racism (Study 2). Taken together, these results provide converging evidence that social representations of history function as significant sociopsychological organizing structures through which negative intergroup attitudes are expressed.

### Theoretical Contributions

Study 1 showed that endorsement of Luso-tropicalism—a hegemonic social representation portraying Portuguese colonialism as uniquely benevolent and non-racist—predicted higher levels of biological and cultural racism, over and above age and education. This finding aligns with previous research demonstrating that Luso-tropicalist beliefs are associated with more negative intergroup attitudes in Portugal (Madeira et al., 2023; Valentim & Heleno, 2018) and extends this literature by situating Luso-tropicalism explicitly within the broader framework of Social Representations of History. In this sense, the results support the claim that hegemonic representations of the past are not merely symbolic narratives, but ideological resources that shape how social hierarchies and intergroup relations are perceived in the present.

Importantly, the Portuguese case illustrates a paradoxical dynamic: a social representation emphasizing national exceptionalism and moral non-racism coexists with, and arguably sustains, the very forms of racism it symbolically denies. This pattern resonates with research on aversive racism, which shows that individuals may strongly endorse egalitarian values while simultaneously engaging in discriminatory behavior (Dovidio & Gaertner, 2004). However, Luso-tropicalism differs from aversive racism in a crucial respect: it is historically embedded in a national narrative that glorifies colonialism and naturalizes power asymmetries between groups. As such, the positive group-image it promotes is not merely a byproduct of binary and asymmetric ingroup-outgroup/dominant-subordinate terms, but historically anchored (Dixon et al., 2020), and especially consequential for forming outgroup attitudes.

Study 2 extended these findings by experimentally testing the causal impact of representations of colonial history. Drawing on educational material, the manipulation operationalized a positive, benevolent colonial narrative that reflects discursive elements commonly found in Portuguese history textbooks (positive condition) and a negative, counter narrative. Consistent with the hypothesized, exposure to a negative representation of colonial history, emphasizing violence, exploitation, and historical wrongdoing, led to lower endorsement of biological racism compared to the pooled positive and control condition. This finding is theoretically significant. Biological racism constitutes an overt form of prejudice that legitimizes social hierarchies through presumed innate differences between groups and has been identified as a particularly consequential predictor of intergroup conflict and discrimination (Vala et al., 2009; Dovidio et al., 2002).

As Howarth (2006) argues, dominant social representations are sustained not only through what is said, but also through what is silenced. In Portugal, benevolent narratives of colonialism widely spread by means of propaganda during the dictatorial regime. Institutionalized through education, such narratives incorporate and normalize attitudes that consequently help people to make sense of the world. The negative representation introduced in Study 2 could function as a counter-narrative that makes historical violence salient, thereby weakening the expression of biologically racist beliefs. We found that a mere exposure to historical wrongdoings carried out by the Portuguese and the hardships endured by people under former colonial rule was sufficient to lower endorsement of biological racism that currently seems to be on the rise again in Portugal (Vala & Pereira, 2018).

Beyond demonstrating this counter-representational effect, the present research makes a specific contribution by linking educational narratives to the reproduction and legitimation of these representations. Across several European contexts, research on history textbooks has documented a gradual shift toward more critical accounts of colonialism, while simultaneously showing the persistence of benevolent and ingroup-favoring narratives that rarely incorporate counter-representations challenging hegemonic interpretations of the past (Van Nieuwenhuyse & Valentim, 2018; Van Nieuwenhuyse, Bentrovato, & Valentim, 2026). For example, studies in Spain reveal that history textbooks continue to present a ‘rosy’ depiction of the colonial past (Pousa & López-Facal, 2013; Brescó de Luna, 2018), while in Belgium colonial history is still predominantly framed from a Western perspective (Van Nieuwenhuyse, 2018). In Portugal, research similarly shows that contemporary textbooks largely maintain benevolent representations of Portuguese colonialism (Valentim & Miguel, 2018). The present study adds causal evidence to this line of research, showing direct pathways from historical representations to racist endorsement.

In line with the historical charter framework (Liu & Hilton, 2005), no differences emerged between the positive representation and the control condition. This pattern suggests that positive representations of colonialism function as historical charters: rather than introducing new information, they ingrain already familiar and widely shared understandings of the national past, embedding baseline levels of attitudinal endorsement that mirror those observed in a neutral condition. However, these null findings should be interpreted with caution as nonsignificant p values should not be used to support the null hypothesis. Specifically, a lack of statistical significance may reflect a lack of power to detect a true effect rather than the actual absence of one (Aczel et al., 2018).

The absence of experimental effects for cultural racism further underscores the differentiated ways in which historical representations relate to distinct forms of prejudice. While biological racism responded to counter-representational exposure, cultural racism remained largely unaffected by the manipulation. This asymmetry is consistent with the idea that contemporary norms more strongly condemn biologically based racism, whereas culturally framed hierarchies may be more deeply normalized and less readily challenged by short-term exposure to historical information (Vala & Perreira, 2018). Rather than undermining the central argument, this pattern highlights the importance of distinguishing between forms of racism when examining the attitudinal consequences of historical representations.

## Future Research and Limitations

The present research opens several avenues for future work on the mechanisms through which social representations of history predict intergroup attitudes. To our knowledge, this is the first study conducted to experimentally manipulate representations of colonial history using textbook-based materials to assess racist attitudes. While this approach offers high ecological relevance, it also represents only a first step toward understanding how educational narratives foster intergroup attitudes.

Textbooks do not merely transmit historical facts, but institutionalized norms and values that guide meaning-making processes and contribute to students’ understanding of intergroup dynamics (Fuchs & Bock, 2018). Future research would therefore benefit from examining educational materials in more ecologically valid contexts, such as classroom-based studies, longitudinal designs, or the use of extended textbook excerpts. Combining experimental and non-experimental approaches in educational settings would allow for a more fine-grained understanding of how historical narratives are internalized and how they influence intergroup attitudes over time. Future studies could more systematically compare dominant hegemonic narratives with explicitly articulated counter-narratives, using larger samples and diverse educational contexts.

Several limitations of the present study should also be acknowledged. First, the assessment of cultural racism differed across studies, and in Study 2 cultural racism was measured using a single item. Although single-item measures can be useful in certain contexts, they may compromise construct validity and reliability when assessing complex ideological constructs such as cultural racism (Diamantopoulos et al., 2012). Future research should employ multi-item measures to capture the multidimensional nature of racism more comprehensively and should consider extending the range of outcome variables to include other indicators of intergroup relations, such as policy support, empathy, or social distance.

Second, the experimental manipulation relied on excerpts from a history textbook published in 2019. While recent work suggests that Portuguese textbooks have undergone minor revisions since then, existing evidence indicates that benevolent representations of colonialism and the omission of colonial violence remain largely intact (e.g., Meuer, Valentim, & Forte, 2026). Nevertheless, the use of a textbook version published several years prior constitutes a limitation, and future research would benefit from employing the most recent educational materials to assess whether emerging changes in textbook narratives alter the impact of historical narratives on attitudes.

Finally, Study 2 did not assess participants’ pre-existing endorsement of Luso-tropicalist beliefs. Although the experimental design allows for causal inference regarding the effects of exposure to contrasting historical narratives, it does not account for individual differences in ideological predispositions. Prior beliefs are likely to shape how historical information is processed, with individuals tending to accept information that confirms their worldview while resisting information that challenges it (Kunda, 1990). Previous research shows that exposure to negative historical information can elicit defensive responses aimed at protecting a positive ingroup image (Bilewicz, Imhoff, & Wójcik, 2013; Bilewicz, 2016). In the present context, participants with stronger Luso-tropicalist beliefs may have responded differently to positive versus negative representations of Portuguese colonial history. Assessing these beliefs prior to exposure would allow future studies to examine moderation effects and to identify which individuals are more susceptible—or resistant—to dominant versus critical historical narratives.

## Conclusion

The present research advances the study of Social Representations of History by demonstrating how dominant social representations of colonialism are implicated in the reproduction of racist beliefs (Study 1). By adopting a historical lens on intergroup relations, this work shows that colonial beliefs, specifically Luso-tropicalism, continue to function as meaningful sociopsychological frameworks through which social hierarchies are perceived and legitimized.

Across two complementary studies, the findings converge on a clear pattern. Endorsement of Luso-tropicalist beliefs was associated with higher levels of both biological and cultural racism, underscoring the role of benevolent colonial representations in sustaining racialized attitudes. Moreover, experimental evidence showed that exposure to a critical representation of Portuguese colonial history reduced endorsement of biological racism relative to exposure to the combined benevolent-neutral condition.

Importantly, this research highlights the educational domain as an institutional context in which social representations of history are reproduced and transmitted. By drawing directly on textbook-based material, the study provides causal evidence that educational narratives do not merely reflect collective memory but actively shape intergroup attitudes. In doing so, it positions schooling as a key site where dominant historical representations can either reinforce or challenge racialized belief systems.

Taken together, these findings contribute to a growing body of work demonstrating that how societies remember and narrate their past has tangible consequences for present-day intergroup relations. By empirically linking historical representations, institutionalized narratives, and racial attitudes within a single framework, the present study highlights the importance of critically examining the narratives through which colonialism continues to be told.

## Data Availability

The anonymized dataset, all materials, and ethical approval (including manipulation texts and survey instruments) are openly available at [https://osf.io/6hefp/?view_only=7f18ab34b63a4e8d9d94c83591c120d8].
